# Oriented attachment of V_NAR_ proteins, *via* site-selective modification, on PLGA–PEG nanoparticles enhances nanoconjugate performance†
†Electronic supplementary information (ESI) available. See DOI: 10.1039/c9cc02655j


**DOI:** 10.1039/c9cc02655j

**Published:** 2019-05-29

**Authors:** João C. F. Nogueira, Michelle K. Greene, Daniel A. Richards, Alexander O. Furby, John Steven, Andrew Porter, Caroline Barelle, Christopher J. Scott, Vijay Chudasama

**Affiliations:** a Department of Chemistry , University College London , London , UK . Email: v.chudasama@ucl.ac.uk; b Centre for Cancer Research and Cell Biology , School of Medicine , Queen's University Belfast , Belfast , UK . Email: c.scott@qub.ac.uk; c Department of Materials , Imperial College London , London , UK; d Institute of Medical Science , University of Aberdeen , Aberdeen , UK; e Elasmogen Ltd , Aberdeen , UK . Email: caroline.barelle@elasmogen.com; f Research Institute for Medicines (iMed.ULisboa) , Faculty of Pharmacy , Universidade de Lisboa , Lisbon , Portugal

## Abstract

Herein we report the construction of a nanoparticle-based drug delivery system which targets a key regulator in tumour angiogenesis. We exploit a Variable New Antigen Receptor (V_NAR_) domain, conjugated using site-specific chemistry, to direct poly lactic acid-*co*-glycolic acid–polyethylene glycol (PLGA–PEG) nanoparticles to delta like canonical Notch ligand 4 (DLL4). The importance of site-specific chemistry is demonstrated.

## Abstract 2

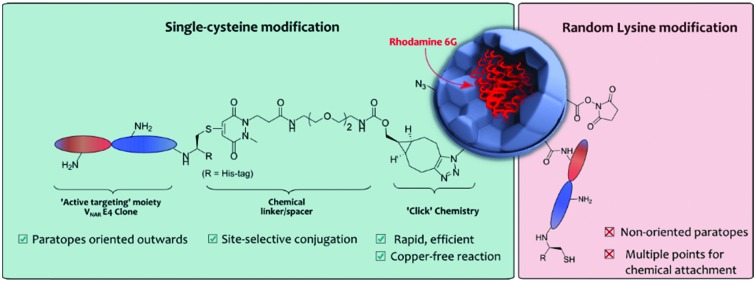
Conjugation of Variable New Antigen Receptors (V_NARs_) to PLGA–PEG nanoparticles in a site-selective manner provides superior nanoparticle–protein constructs

## 

Over the last few years the utilisation of nanotechnology within biomedicine, particularly in the field of targeted therapy, has grown considerably.^1^,^2^ Nanoscale materials can cross biological barriers including the blood–brain barrier,^3^ transit in and out of blood vessels^4^ or even passively penetrate the cell membrane by different mechanisms (*e.g.* endocytosis).^5^ Furthermore, nanoparticles (NPs) are able to safely transport different types of cargo, such as molecular imaging agents or cytotoxic drugs in high quantities.^6^ Most recently, NPs have also been combined with targeting proteins, such as antibodies or antibody fragments, to enable the targeting of disease biomarkers in a concept tagged “active targeting”.^7^ Although antibodies have been employed for the generation of actively targeted NPs, the clinical application of the resultant nanoconjugates has remained rare. This is due in part to suboptimal methods for the appendage of proteins to NPs.^8^ For instance, many protocols employ random conjugation methods, such as electrostatic adsorption of proteins to NP surfaces, to achieve targeting.^9^ This method is mediated by the surface charge of antibodies, which varies significantly from antibody to antibody, and can lead to significant batch-to-batch variability of resulting nanoconjugates and poor stability.^10^,^11^ To overcome these issues, methods which rely on chemically stable covalent bonds to graft antibodies to NPs have been developed; these methods are reported to confer better stability *in vivo*.^12^ Early developers focused on covalently attaching antibodies (*via* surface lysine residues) to NP *via* carbodiimide chemistry.^13^–^15^ However, this is sub-optimal as it usually presents low reaction efficiencies in aqueous conditions due to competing hydrolysis.^16^ Conjointly, the modification of lysine residues affords very little control over the orientation of the antibody targeting ligands on the NP surface, limiting antigen-binding and overall target avidity. We recently demonstrated the importance of controlled chemical ligation for successful nanoconjugate performance, particularly in the context of target affinity.17*a* This study showed that using site-selective chemistry to conjugate Trastuzumab antibody fragments (*i.e.* F(ab)s) to PLGA–PEG NPs resulted in superior antigen binding when compared to using classical lysine-based approaches.17*a*

There is a growing trend in NP construction towards utilising small antibody fragments rather than full antibodies as active targeting ligands.^18^ Smaller size permits a greater loading on NP surfaces, whilst maintaining the antigen-binding capability of a full antibody, and absence of a fragment crystallisable (Fc) region can reduce Fc-mediated immunogenicity.^18^–^20^ Methods of generating antibody fragments are well described in literature, *e.g.* F(ab)′ and F(ab) (both *ca.* 50 kDa) can be generated through enzymatic digestions,^18^ and have been successfully employed in the formation of nanoconjugates for the treatment of multiple diseases.17a,^21^,^22^ However, there is a drive to find alternative proteins that share the same specific binding properties of antibodies but that are of an even smaller size. Shark Variable New Antigen Receptors (V_NARs_), at only *ca.* 11 kDa, are seen as an attractive alternative to current traditional antibodies and their fragments as they hold many advantages such as higher stability, solubility and low cost of manufacturing, while upholding high specificity to bind to novel or cryptic epitopes.^23^–^25^ Most relevantly, the simple V_NAR_ molecular architecture presents a versatile platform for re-formatting and engineering. For instance, V_NARs_ have been previously engineered to increase their solubility and refolding ability^26^ and also to bind to many different antigens with high specificity.^27^ Also, V_NARs_ are reported to tolerate extreme pH values (down to pH 1.5) and high temperature conditions. These advantages have allowed the facile generation of a plethora of new biologics.^28^ However, their applications within nanotechnology remain to be exploited.

Herein is presented a novel PLGA–PEG NP–V_NAR_ conjugate that targets the inhibition of endothelial sprouting and proliferation (processes involved in tumour angiogenesis).^29^ We hypothesised that by applying a highly-directed chemical strategy for decorating nanocarriers with V_NAR_ ligands, the overall potential of V_NAR_ directed nanocarriers could be maximised, *i.e.* antigen binding enhancement due to the favourable orientation and large number of proteins packed on the NP surface. To ensure modularity, we created a platform that is amenable to commonly employed and well-tolerated copper (Cu)-free “click” chemistry, namely strain-promoted azide–alkyne cycloaddition (SPAAC) chemistry.

PLGA–PEG polymeric NPs were chosen as the nanocarrier platform due to their well-established ability to effectively hold cargo for controlled drug delivery.17*a* However, we anticipate that the findings of this study could be extrapolated to emerging nanocarriers *e.g.* poly(ε-caprolactone)–PEG, which recent findings have suggested could be ideal drug carriers due to excellent cellular internalization and prolonged blood circulation time.17b,c Owing to our past experience in synthesizing such PLGA–PEG constructs we used a 75 : 25 blend of PLGA-502H : PLGA–PEG-azide to formulate our NP-azide constructs for “click” modification, as these NPs exhibit long-term stability.17*a*

Subsequently, an anti-DLL4 E4 V_NAR_ clone, hereafter referred to as V_NAR_ E4 **6**, was used as a model V_NAR_ targeting moiety. V_NAR_ E4 **6** clone was specifically expressed to recognise and bind to DLL4, a key regulator that activates Notch signaling pathways directly related with early embryonic vascular development in tumor angiogenesis (see ESI,† for details).^30^ An Alanine–Cysteine–Alanine (ACA) sequence was inserted at the C-terminal region, enabling site-selective cysteine modification. Also, this reactive cysteine thiol handle was inserted distal from the antigen binding site, at the N-terminus, in view of maximising orientation benefits and minimising any disturbance of the paratope–epitope interaction. To site-selectively modify V_NAR_ E4 **6** and introduce a strained alkyne moiety we synthesised monobromopyridazinedione **5**. Bromopyridazinediones are selectively reactive towards cysteine-bearing proteins, and bicyclo[6.1.0]nonyne (BCN(endo)) (BCN) is well suited to “click” reactions with azide-bearing NPs in aqueous buffers.^31^ Synthesis of strained alkyne monobromopyridazinedione (PD **5**) proceeded from readily available starting materials in a facile manner over five steps (Scheme 1).^32^ Initially, di-Boc protection of methyl-hydrazine **1** and subsequent Michael addition to *tert*-butyl acrylate yielded hydrazine **2**. Following this, deprotection and dehydration under acidic conditions afforded acid PD **3**. Subsequent esterification of acid **3** with NHS afforded amine-reactive PD **4**. In the final step, an amide coupling between commercially available BCN(endo)–PEG_2_–NH_2_ and PD **4** yielded the desired heterobifunctional PD linker **5** (Scheme 1).

**Scheme 1:**
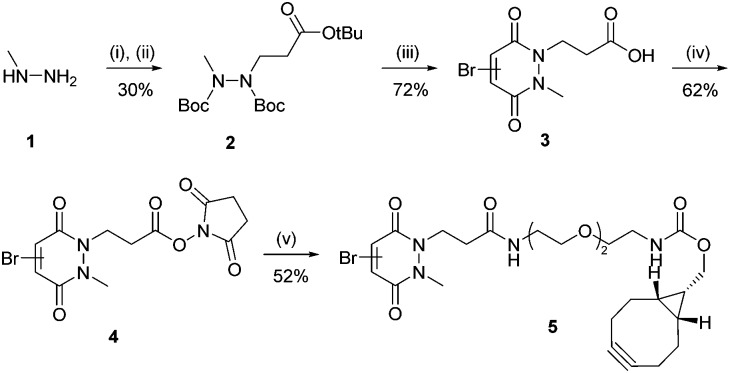
Synthesis route of PD **5**. *Reagents and conditions*: (i) Boc anhydride, i-PrOH, DCM, 21 °C, 16 h; (ii) *tert*-butyl acrylate, i-PrOH, 60 °C, 24 h; (iii) bromomaleic acid, AcOH, reflux, 5 h; (iv) DCC, NHS, THF, 21 °C, 16 h, (v) BCN(endo)–PEG2–NH_2_, MeCN, 21 °C, 16 h.

Bioconjugation of native V_NAR_ E4 **6** with PD **5** was appraised *via* SDS-PAGE and LC-MS, where it was observed that complete conversion only occurred following pre-reduction of V_NAR_ E4 **6** with tris(2-carboxyethyl)phosphine (TCEP). This can be explained by the fact that the single cysteine can be capped with glutathione or even spontaneously form a disulfide dimer in solution. Several optimisation parameters were tuned to obtain pure V_NAR_ conjugate **7** (see ESI,† for details). The optimised conditions for successful site-selective modification comprised pre-incubation of V_NAR_ E4 **6** with TCEP (10 eq.), followed by addition of PD **5** (20 eq.); full reduction and complete conversion were confirmed by LC-MS. To demonstrate the availability of V_NAR_ conjugate **7** to participate in a “click” reaction, the construct was successfully reacted with Alexafluor®-488-N_3_ (Scheme 2, conjugate **8**). Having successfully modified the C-terminal cysteine residue of the V_NAR_ E4 with PD **5**, we next aimed to couple the protein to the surface of polymeric azide NPs in a site-selective manner. Previously, we have shown that similar heterobifunctional linkers can be introduced into antibody F(ab) fragments, thereby facilitating site-specific “click” conjugation to complementary azide-functionalised PLGA–PEG NPs of approximately 200 nm in diameter (Table S1, ESI†).17*a* In concert with this strategy, the strained alkyne functionality of V_NAR_ conjugate **7** was reacted with surface-exposed azide groups on PLGA–PEG-azide NP **10***via* Cu-free SPAAC chemistry to generate E4 functionalised NP (site-selective cysteine modified E4 NP **12**) of 214.2 ± 13.3 nm in size (Fig. 1 and Table S1, ESI†). A benchmark formulation was also included in this study to allow direct comparison of our coupling strategy with carbodiimide chemistry; an established nanoconjugation approach that has been widely implemented in the development of targeted NP modules. Here, amine groups distributed throughout native V_NAR_ E4 **6** (*i.e.* containing no PD **5** linker) were reacted with NHS esters on the surface of PLGA–PEG–NHS NP **9**, with coupling proceeding *via* amide bond formation to generate a nanoconjugate of 197.8 ± 5.1 nm in size (random lysine modified E4 NP **11**). Interestingly, the conjugation efficiency was revealed to be greater for the SPAAC click chemistry when compared to the control; this is in agreement with our previously reported data.17*a* Also, non-targeted NHS and azide NP controls were utilised, of 191.5 ± 5.3 nm and 194.0 ± 2.5 nm in size, respectively (NHS NP **9** and azide NP **10**). To confirm that V_NAR_ E4 still retained its antigen binding affinity after chemical manipulation and nanoconjugation, fluorescently labelled NPs conjugated to V_NAR_ E4, *i.e.***11** and **12**, were incubated with recombinant human DLL4 immobilised on microtiter plates. Dose-dependent binding of NP **12** to DLL4 was observed, with each increment in NP concentration leading to a stepwise enhancement in fluorescence (Fig. 2). Despite similar concentration-dependent binding of NP **11** to DLL4, fluorescence readouts were significantly lower than those observed for NP **12**. The controls showed the binding of non-targeted control NPs (*i.e.* NPs **9** and **10**) to be negligible. Moreover, to confirm that the enhanced binding of NP **12** was not simply due to the higher protein loading on these NP, we next formulated NP **11** and NP **12** to present equal amounts of V_NAR_ E4 **6** and V_NAR_ conjugate **7**, respectively, on their surfaces. Again, DLL4 binding by NP **12** remained superior (Fig. S3, ESI†). Collectively, these findings demonstrate that our novel chemistry may be exploited for the site-specific “click” conjugation of DLL4-targeted V_NARs_ to polymeric NPs, yielding nanoconjugates with superior binding ability than those formulated using conventional methods.

**Scheme 2:**
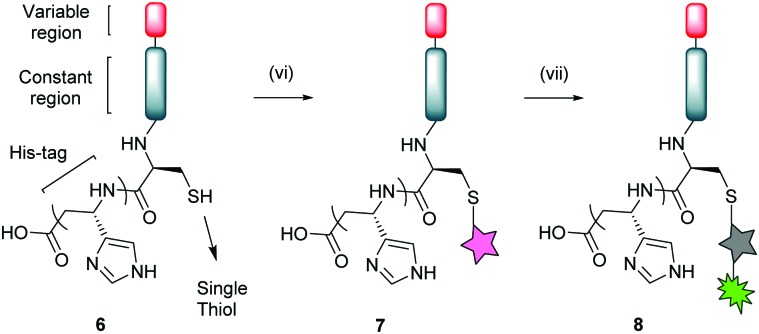
Bioconjugation of V_NAR_ E4 **6** with PD **5** and subsequent “click” reaction with Alexafluor®-488-N_3_. *Reagents and conditions*: (vi) TCEP·HCl (10 eq.), PD **5** (20 eq.), phosphate buffer pH 7.4, 21 °C, 16 h. (vii) Alexafluor®-488-N_3_, phosphate buffer pH 7.4, 21 °C, 5 h.

**Fig. 1:**
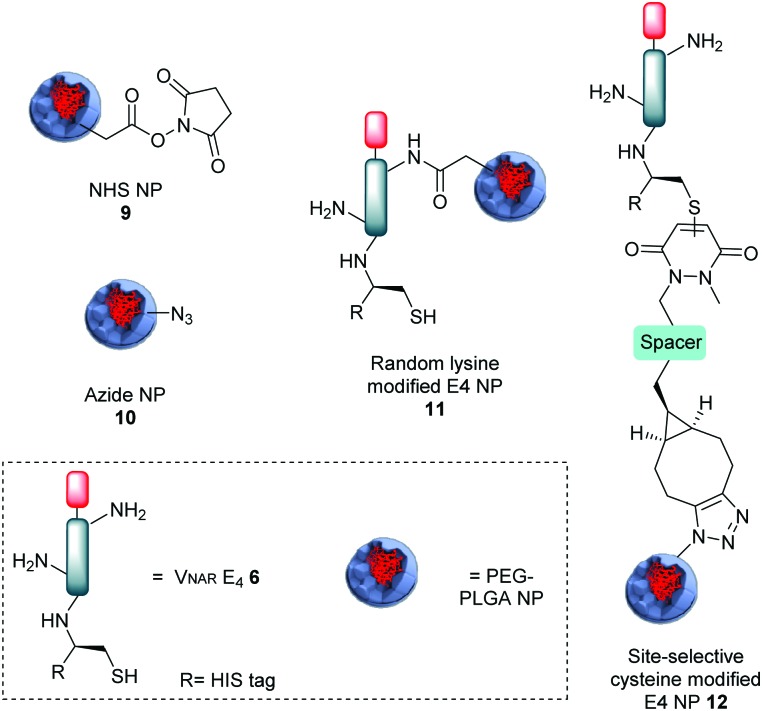
Representation of all nanoformulations tested: NHS NP **9**; azide NP **10**; random lysine modified E4 NP **11** and site-selective cysteine modified E4 NP **12**.

**Fig. 2:**
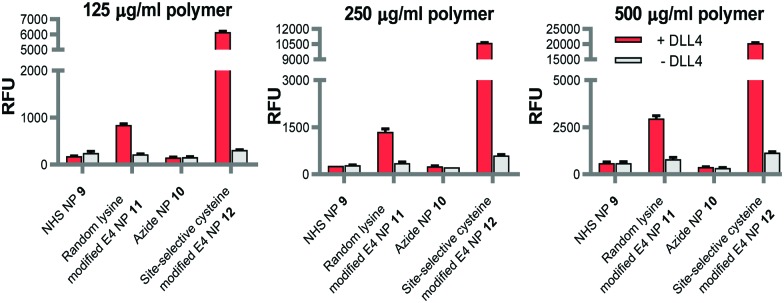
Binding of site-selective cysteine modified E4 NP **12** to DLL4 is greatly enhanced compared to random lysine modified E4 NP **11**. Binding of fluorescently labelled NPs **11** & **12**, and corresponding blank NP controls **9** & **10** (125, 250 and 500 μg polymer mL^–1^) to DLL4 was analysed by modified ELISA. Data expressed as mean ± SEM.

Following this, we sought to verify that the observed enhancement in fluorescence in the above binding assays was attributed to specific interactions between the targeted nanoformulations and the immobilised DLL4 antigen. To do this, the E4 paratopes on the surface of the NP were saturated with an excess of free DLL4 prior to incubation in microtiter plate wells coated with the same antigen. Binding of NP **11** and **12** was inhibited following pre-incubation with DLL4, as evidenced by significantly lower fluorescence readouts for these samples (Fig. 3A). As an alternative approach, both NP **12** and an anti-DLL4 monoclonal antibody were added simultaneously to DLL4-immobilised wells. In these studies, NP binding was progressively impeded with increasing concentrations of competing anti-DLL4 (Fig. 3B). Taken together, these experiments provide robust confirmation that the ability of the NP formulations to bind to DLL4 was dependent upon the surface conjugation of the V_NAR_ proteins.

**Fig. 3:**
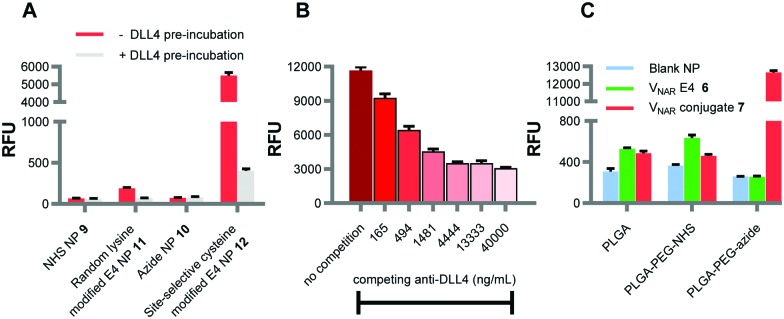
Binding of E4 nanoformulations is DLL4-specific. (A) Binding of fluorescently labelled NP **11**, NP **12** and corresponding blank NP controls **9** and **10** (50 μg polymer mL^–1^) to DLL4 was analysed by modified ELISA ± pre-incubation with DLL4 (10 μg mL^–1^). (B) Binding of fluorescently labelled NP **12** (250 μg polymer mL^–1^) to DLL4 was analysed by modified ELISA ± competition with DLL4 antibody (165–40 000 ng mL^–1^). (C) Enhanced DLL4 binding by NP **12** is dependent upon site-specific “click” coupling. V_NAR_ conjugate **7** and V_NAR_ E4 **6** were incubated with fluorescently labelled NP composed of (1) PLGA-502H, (2) a 75 : 25 blend of PLGA-502H : PLGA-PEG-NHS or (3) a 75 : 25 blend of PLGA-502H : PLGA-PEG-azide. Binding of these nanoformulations and corresponding blank NP controls (500 μg polymer mL^–1^) to DLL4 was analysed by modified ELISA. Data expressed as mean ± SEM.

We next investigated whether the superior binding of site-selective cysteine modified E4 NP **12** was contingent upon both surface display of azide and cysteine modification of the V_NAR_. Various nanoformulations were synthesised by incubating strained alkyne bearing V_NAR_ conjugate **7** with NP comprised solely of PLGA-502H, or a blend of PLGA-502H and either PLGA–PEG–NHS or PLGA–PEG-azide. Binding of these NP to immobilised DLL4 was minimal, except for those formulated *via* “click” coupling of V_NAR_ conjugate **7** to complementary azide-terminated NP (Fig. 3C). Furthermore, native V_NAR_ E4 **6** was also incubated with the above polymeric NP formulations; all three formulations showed only marginal levels of DLL4 binding (Fig. 3C). These findings clearly indicate that the enhanced DLL4 binding activity of NP **12** is not simply mediated *via* non-specific surface adsorption of the V_NAR_ clone. Rather it shows that the presence of the surface-exposed azide and the strained-alkyne modified V_NAR_ are critical determinants of nanoconjugate performance. This confirms the site-selectivity and importance of covalent conjugation.

As a final set of studies, we explored the impact of different loadings of strained alkyne bearing V_NAR_ conjugate **7** on the surface of the NP. As expected, incremental addition of V_NAR_ conjugate **7** resulted in cumulative improvements in DLL4 binding. Interestingly, fluorescence levels plateaued upon adding >2 nanomoles of V_NAR_ conjugate **7** per milligram of polymer, suggesting epitope saturation (see Fig. S4, ESI†).

In conclusion, this study showcases a new modular method for attaching V_NAR_ proteins onto the surfaces of PLGA–PEG polymeric NPs *via* “click” chemistry. A novel heterobifunctional PD linker was designed to enable site-selective cysteine modification of a V_NAR_ clone that was attached to an azide decorated NP *via* SPAAC conjugation. This NP–V_NAR_ construct, with orientated protein presentation on the NP surface, showed favourable properties in terms of target binding when compared to traditional NP–protein conjugation chemistries.

This work was partially funded through a US-Ireland R&D Partnership grant (STL/5010/14, MRC grant MC_PC_15013). JCFN is funded by the EU's Horizon 2020 programme under Marie-Curie grant agreement 675007. We acknowledge UCL Chemistry Mass Spectrometry Facility (Dr K. Karu/Dr X. Yang).

## Conflicts of interest

There are no conflicts to declare.

## Supplementary Material

Supplementary information
