# Supplementary material for: Oriented attachment of VNAR proteins, via site-selective modification, on PLGA–PEG nanoparticles enhances nanoconjugate performance
Source: Chem Commun (Camb). 2019 May 29;55(53):7671–4. doi: 10.1039/c9cc02655j (PMC6873773; doi:10.1039/c9cc02655j)
Supplement: Supplementary file 1 [file CC-055-C9CC02655J-s001.pdf]

Electronic Supplementary Material (ESI) for ChemComm.
This journal is © The Royal Society of Chemistry 2019

Electronic Supplementary Information

Oriented attachment of V_{NAR} proteins, *via* site-selective modification, on PLGA-PEG nanoparticles enhances nanoconjugate performance

João C. F. Nogueira,[a] Michelle K. Greene,[b] Daniel A. Richards,[a,c] Alexander O. Furby,[a] John Steven,[d,e] Andrew Porter,[d,e] Caroline Barelle,*[d,e] Christopher J. Scott *[b] and Vijay Chudasama *[a,f]

a. Department of Chemistry, University College London, 20 Gordon Street, WC1H 0AJ, London, UK.
b. Centre for Cancer Research and Cell Biology, School of Medicine, Queen's University Belfast, Belfast, UK.
c. Department of Materials, Imperial College London, London, UK
d. Biochemistry Department, University of Aberdeen, Aberdeen, UK.
e. Elasmogen Ltd, Aberdeen, UK.
f. Research Institute for Medicines (iMed.ULisboa), Faculty of Pharmacy, Universidade de Lisboa, Lisbon, Portugal

General Experimental

All chemical reagents were purchased from Sigma Aldrich, Alfa Aesar, Thermo Fisher and Acros. Compounds and solvents were used as received. Petrol refers to petroleum ether (b.p. 40–60 °C). All reactions were carried out under positive pressure of argon, unless stated otherwise, and were monitored using thin layer chromatography (TLC) on pre-coated silica gel plates (254 μm). Detection was by UV (254 nm and 365 nm) or chemical stain ($KMnO_4$, ninhydrin, iodine). The term *in vacuo* refers to solvent removal using Büchi rotary evaporation between 15–60 °C, at approximately 10 mm Hg. Flash column chromatography was carried out with pre-loaded GraceResolv flash cartridges on a Biotage Isolera Spektra One flash chromatography system. ^{1}H NMR spectra were obtained at 600 MHz. ^{13}C NMR spectra were obtained at 150 MHz. All results were obtained using Bruker NMR instrument Avance III 600. All samples were run at the default number of scans and at 21 °C. Chemical shifts (δ) for ^{1}H NMR and ^{13}C NMR are quoted relative to residual signals of the solvent on a parts per million (ppm) scale. Where amide rotamers are the case, and when possible, every rotamer has been assigned for chemical shifts and have been considered for integration calculations. Where regioisomers are reported, each isomer was characterised individually, where possible. Coupling constants (J values) are reported in Hertz (Hz) and are reported as $J_{H\text{-}H}$ couplings. The multiplicity of each signal is indicated as s-singlet, d-doublet, t-triplet and m-multiplet (*i.e.* complex peak obtained due to overlap). Infrared spectra were obtained on a Perkin Elmer Spectrum 100 FTIR spectrometer operating in ATR mode. Mass spectra were obtained, for synthetic products, from the UCL mass spectroscopy service on either a Thermo Finnigan MAT900Xp (EI and CI) or Waters LCT Premier XE (ES) mass spectrometer or obtained by the EPSRC UK National Mass Spectrometry Facility (NMSF), Swansea. Melting points were measured with Gallenkamp apparatus and are uncorrected.

Bioconjugation general remarks

Conjugation experiments were carried out in standard polypropylene Eppendorf® safe-lock tubes (2.0 mL) at atmospheric pressure. All buffer solutions were prepared with doubly deionized water and filter-sterilized. Phosphate-buffered saline (PBS) was 12 mM phosphates, 140 mM NaCl at pH 7.4. Ultrafiltration was carried out in Amicon® Ultra-4 Centrifugal Filter Units with a molecular weight cut-off (MWCO) of 5 kDa or in Vivaspin® 500 centrifugal concentrators (5 kDa MWCO). Centrifugation was carried out on an eppendorf 5415R fixed angle rotor centrifuge operating at 14000 rcf at 20 °C or in an eppendorf 5810 swing-bucket rotor centrifuge operating at 3220 rcf at 20 °C. Purification by size exclusion chromatography (SEC) was carried out on an ÄKTA FPLC system (GE Healthcare), equilibrated in PBS. Detection was by absorption at 280 nm.

SDS-PAGE

Non-reducing glycine-SDS-PAGE at 18% acrylamide were performed following standard lab procedures. A 6% stacking gel was used and a broad-range MW marker (10–250 kDa, Prestained PageRuler Plus Protein Standards, ThermoScientific) was co-run to estimate protein weights. Samples (5 μL at 33 μM) were mixed with loading buffer (2 μL, composition for 5 × SDS: 1 g SDS, 3 mL glycerol, 6 mL 0.5 M Tris buffer pH = 6.8, 2 mg bromophenol blue in 10 mL), heated at 75 °C for 5 mins, and centrifuged at 14000 rcf for 1 min. Samples were subsequently loaded into the wells in a volume of 5 μL. All gels were run at 80 V for 15 mins, then 150 V until complete. Gels were stained using a Coomasie stain.

UV-Vis spectroscopy

UV-Vis spectroscopy was used to determine protein concentrations, using a Varian Cary 100 Bio UV–Visible spectrophotometer operating at 21 °C. Sample buffer was used as blank for baseline correction. Extinction coefficient of V_{NAR} E4 is ε_{280} = 24075 M^{-1} cm^{-1}.

Determination of Protein Masses by Liquid Chromatography Mass Spectrometry (LC-MS)

LC-MS was performed on protein samples using a Waters Acquity uPLC connected to Waters Acquity Single Quad Detector (SQD). Column: Hypersil Gold C4, 1.9 μm, 2.1 × 50 mm. Wavelength: 254 nm. Mobile Phase: 95:5 Water (0.1% Formic Acid): MeCN (0.1% Formic Acid) Gradient over 4 min (to 5:95 Water (0.1% Formic Acid): MeCN (0.1% Formic Acid). Flow Rate: 0.6 mL/min. MS Mode: ES+. Scan Range: m/z = 500–2000. Scan time: 0.25 s. Data obtained in continuum mode. The electrospray source of the MS was operated with a capillary voltage of 3.5 kV and a cone voltage of 50V. Nitrogen was used as the nebulizer and desolvation gas at a total flow of 600 L/h. Total mass spectra for protein samples were reconstructed from the ion series using the MaxEnt1 algorithm pre-installed on MassLynx software.

Preparation of nanoformulations

Unless otherwise indicated, DLL4-targeted nanoformulations were synthesised by adding equimolar amounts (0.25, 0.5, 1, 2 or 4 nanomoles per milligram of polymer) of V_{NAR} E4 **6** or V_{NAR} conjugate **7** to rhodamine 6G-loaded NP composed of either: (1) PLGA-502H; (2) a 75:25 blend of PLGA-502H:PLGA-PEG-NHS; or (3) a 75:25 blend of PLGA-502H:PLGA-PEG-azide. Full details on the methods used for NP fabrication and surface functionalisation are as previously described.[1]

Characterisation of nanoformulations

Nanoparticle size, polydispersity index and zeta potential were analysed using a NanoBrook Omni (Brookhaven Instruments Corp), after resuspension at 200 µg polymer/mL in PBS. A Micro BCA protein assay kit (Thermo Scientific) was used to quantify V_{NAR} loading on each nanoformulation as previously described.[1]

Modified enzyme-linked immunosorbent assay (modified ELISA)

Binding of NP to human DLL4 Fc chimera protein (Sino Biological) was evaluated as previously described.[1] For targeting specificity studies, NP were pre-incubated with DLL4 Fc chimera (10 µg/mL) in free format for 30 mins at room temperature, prior to addition to microtiter plates containing the same protein in immobilised format. Alternatively, targeting specificity was assessed through simultaneous addition of NP and a competing anti-DLL4 monoclonal antibody (0-40 µg/mL; R&D systems) to antigen-coated plates.

E4 clone data

V_{NAR} Clone E4 ACA version design and synthesis

The Alanine Cysteine Alanine (ACA) insertion in the C-terminal region of V_{NAR} E4 clone was designed in silico using DNASTAR® software. Flanking the V_{NAR} gene sequence *Nco*I and *EcoR*I restriction endonuclease sites as well as a poly-histidine (6×histidine) tag were incorporated to facilitate cloning into a prokaryotic expression and IMAC protein purification and immunodetection respectively (Figure S1). The in-house designed gene (Figure S1) was synthesised by PCR amplification of the V_{NAR} E4 DNA template using oligonucleotides #249_Forward and #256_Reverse using 2×Phusion High-Fidelity PCR Master Mix (New England Biolabs).

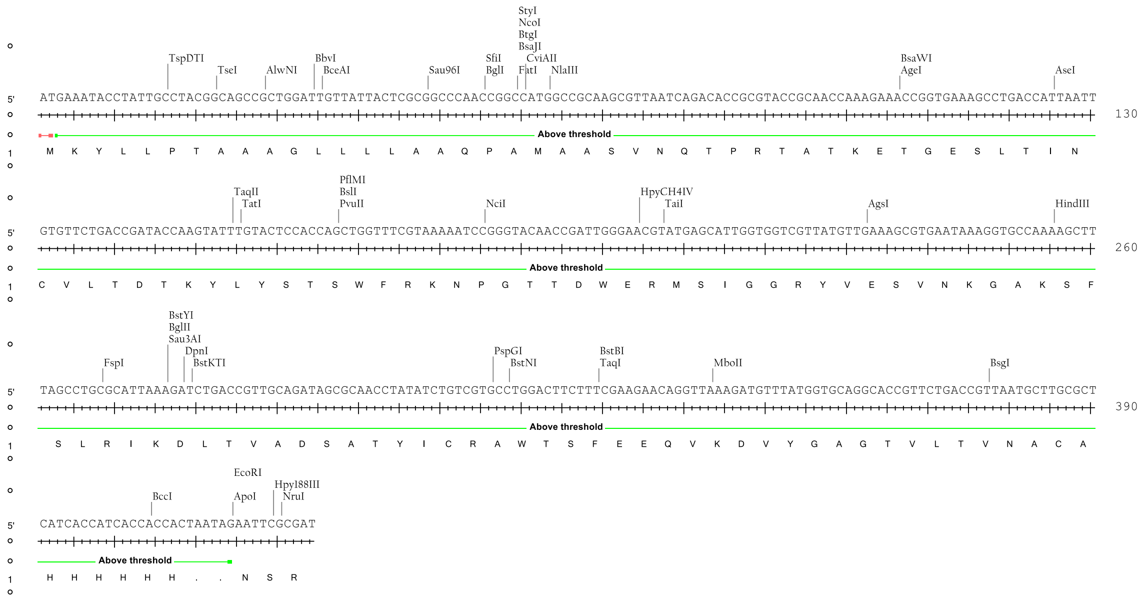

Figure S1. A detailed in-silico sequence of clone E4 highlighting the pelB leader sequence, restriction sites, ACA insertion, and poly histidine tag.

Cloning V_{NAR} E4 ACA into pIMS147 and *E. coli* TG1 Transformation

This was achieved by digestion of the pIMS147 and E4 ACA amplicon with *Nco*I and *EcoR*I restriction endonucleases. Digested plasmid and insert DNA were ligated using T4 ligase enzyme (New England Biolabs). Resultant ligated mixture was transformed into electrocompetent *E. coli TG1* and plated out on selective growth media 2×TY-AG agar plates. After overnight growth, colonies were selected and grown overnight in 2×TY-AG broth. Plasmid DNA was prepared from overnight cultures using a Qiagen Miniprep DNA kit and samples sent for sequencing. A clone with confirmed correct sequence was selected and used for protein expression.

Soluble V_{NAR} Protein Expression and Purification

1 L of 2×TY-AG growth media was inoculated with a 5 mL overnight culture of V_{NAR} E4. The inoculated culture was grown overnight at 37 °C, 250 rpm, followed by centrifugation at 6000 x g for 10 mins. The resulting cell paste was resuspended in 1 L of TB-Amp media, and grown at 30 °C, 250 rpm for 1 h. Protein expression induction was initiated by adding IPTG to the media at a final concentration of 1 mM and grown for a further 4 h at 30 °C, 250 rpm.

Cells were centrifuged at 6000 × g for 30 mins, and supernatant discarded. The cell pellet was resuspended in 100 mL of osmotic shock buffer (20 % w/v sucrose, 100 mM Tris HCl pH 7.6, 5 mM EDTA). Osmotic shock sample was placed on a rolling platform mixer at 50 rpm, for 15 mins at room temp. Finally, 100 mL of 5 mM $MgCl_2$ was added to the sample and mixing continued for a further 15 mins.

Sample was centrifuged at 15000 × g for 30 mins, 4 °C and supernatant was collected. A 1/10 volume of 10×PBS was added to the resulting supernatant.

IMAC purification of expressed his-tagged protein was performed by addition of 3 mL of Ni^{2+} charged IMAC resin to the supernatant then the resultant suspension mixed for 1 h on a roller mixing platform at 50 rpm. Post batch binding, the IMAC resin was collected in a column and unbound proteins passed through the column under gravity. Nickel resin was washed with 50 mL of 1×PBS containing 10 mM Imidazole. Bound protein was eluted with 10 mL of 250 mM Imidazole, pH 8.

The batch IMAC purified protein was subject to a second round of purification using Anion Exchange chromatography. The eluted sample was buffer exchanged to 30 mM Tris HCl, pH 9.5, and loaded onto the AKTA Prime Chromatography system with a gradient elution using 20 column volumes of 1 M NaCl 30 mM Tris HCl pH 9.5. Analysis of protein samples across the eluted peaks by SDS PAGE confirmed fractions contained E4 ACA protein. Fractions were then polled, and buffer exchanged to 1×PBS, pH 7.4. Protein concentration was determined using the A280 spectrophotometer.

Notes:

1. 2×TY: In 1 L de-ionised water, dissolve 16 g, 10 g and 5 g of Tryptone, Yeast extract and NaCl respectively, then autoclave.
2. 2×TY-AG: 2×TY, 100 µg/mL ampicillin and 2 % (w/v) glucose.

3. Terrific Broth (TB)-Amp: In 900 L de-ionised water, dissolve 12 g and 24 g of Tryptone and Yeast extract respectively. Add 4 mL of glycerol, then autoclave. To autoclaved media, add 100 mL of sterile Phosphate salts (In 500 mL de-ionised water, dissolve 11.55 g and 62.7 g of Potassium phosphate monobasic-KH_2PO_4 and Potassium phosphate dibasic-K_2HPO_4 respectively). Add µg/mL ampicillin final concentration.

E4 Nucleotide sequence

GCAAGCGTTAATCAGACACCGCGTACCGCAACCAAAGAAACCGGTGAAAGCCTGACCAT
TAATTGTGTTCTGACCGATACCAAGTATTTGTACTCCACCAGCTGGTTTCGTAAAAATCC
GGGTACAACCGATTGGGAACGTATGAGCATTGGTGGTCGTTATGTTGAAAGCGTGAATA
AAGGTGCCAAAAGCTTTAGCCTGCGCATTAAAGATCTGACCGTTGCAGATAGCGCAACC
TATATCTGTCGTGCCTGGACTTCTTTCGAAGAACAGGTTAAAGATGTTTATGGTGCAGGC
ACCGTTCTGACCGTTAAT

E4 Amino acid sequence

ASVNQTPRTATKETGESLTINCVLTDTKYLYSTSWFRKNPGTTDWERMSIGGRYVESVNKGA
KSFSLRIKDLTVADSATYICRAWTSFEEQVKDVYGAGTVLTVN

E4 ACA nucleotide sequence with pelB leader sequence

ATGAAATACCTATTGCCTACGGCAGCCGCTGGATTGTTATTACTCGCGGCCCAACCGGCC
ATGGCCGCAAGCGTTAATCAGACACCGCGTACCGCAACCAAAGAAACCGGTGAAAGCCT
GACCATTAATTGTGTTCTGACCGATACCAAGTATTTGTACTCCACCAGCTGGTTTCGTAA
AAATCCGGGTACAACCGATTGGGAACGTATGAGCATTGGTGGTCGTTATGTTGAAAGCG
TGAATAAAGGTGCCAAAAGCTTTAGCCTGCGCATTAAAGATCTGACCGTTGCAGATAGC
GCAACCTATATCTGTCGTGCCTGGACTTCTTTCGAAGAACAGGTTAAAGATGTTTATGGT
GCAGGCACCGTTCTGACCGTTAATGCTTGCGCTCATCACCATCACCACCAC

E4 ACA amino acid sequence with pelB leader sequence

MKYLLPTAAAGLLLLAAQPAMAASVNQTPRTATKETGESLTINCVLTDTKYLYSTSWFRKN
PGTTDWERMSIGGRYVESVNKGAKSFSLRIKDLTVADSATYICRAWTSFEEQVKDVYGAGT
VLTVNACAHHHHHH

Oligo #249

CTCGCGGCCCAACCGG*CCATGG*CCGCAAGCGTTAATCAGACACCGCG

Oligo #256

ATCGC*GAATTC*TATTAGTGGTGGTGATGGTGATGAGCGCAAGCATTAACGGTCAGAACGG
TGCCTGCACC

NB: NcoI and EcoRI sites in oligos #249 and #256 are underlined and italicised.

Synthesis of compounds

Di-*tert*-butyl 1-methylhydrazine-1,2-dicarboxylate[2]

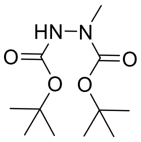

To a stirring solution of *N*-methylhydrazine **1** (157 mg, 3.42 mmol) in *i*-PrOH (4.3 mL) was added dropwise di-*tert*-butyl dicarbonate (1.6 g, 7.5 mmol, pre-dissolved in CH_2Cl_2 (3.4 mL)) over 20 min. The mixture was then stirred for 16 h at 21 °C. After this time, solvent was removed *in vacuo* and purification by flash column chromatography (20% Et_2O/petrol) yielded di-*tert*-butyl 1-methylhydrazine-1,2-dicarboxylate (407 mg, 2.05 mmol, 60%) as a white solid: m.p. 58–62 °C (*lit m.p.* 54–56 °C). ^{1}H NMR (600 MHz, $CDCl_3$, rotamers) δ 6.45 (br s, 0.5H), 6.20 (br s, 0.5H), 3.09 (s, 3H), 1.52-1.44 (m, 18H); ^{13}C NMR (150 MHz, $CDCl_3$, rotamers) *δ* 171.2 (C), 155.9 (C), 81.3 (C), 60.4 (CH_3), 28.3 (CH_3); IR (solid) 3316, 2978, 2932, 1701 cm^{-1}.

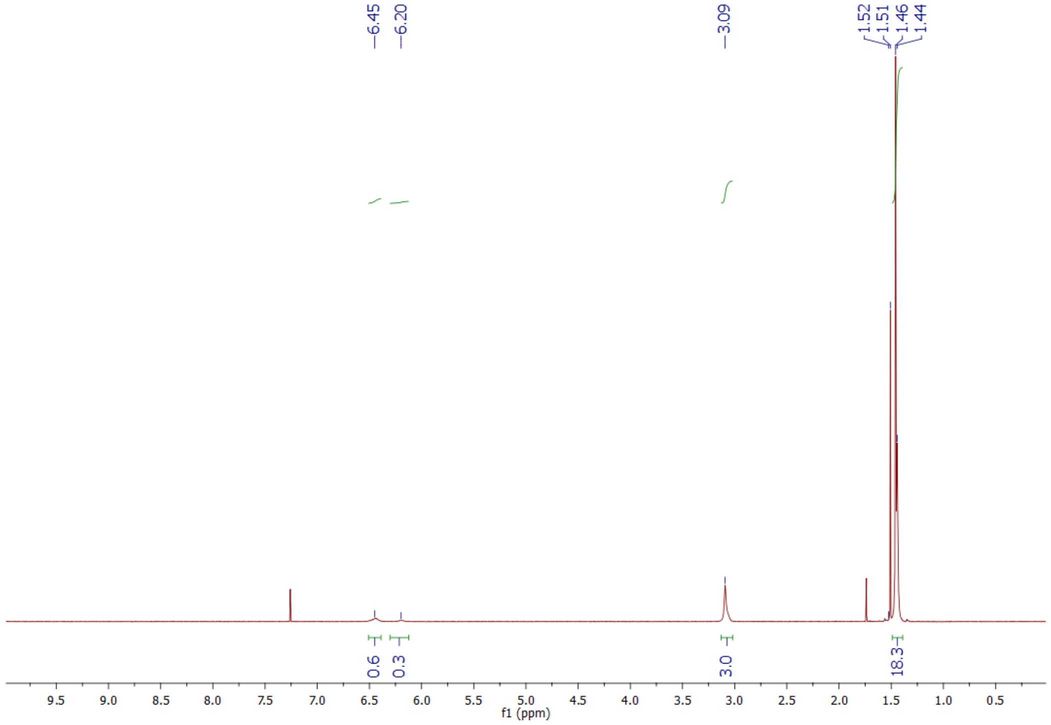
6.45
6.20
3.09
1.52
1.51
1.46
1.44
0.6
0.3
3.0
18.3
9.5
9.0
8.5
8.0
7.5
7.0
6.5
6.0
5.5
5.0
4.5
4.0
3.5
3.0
2.5
2.0
1.5
1.0
0.5
f1 (ppm)

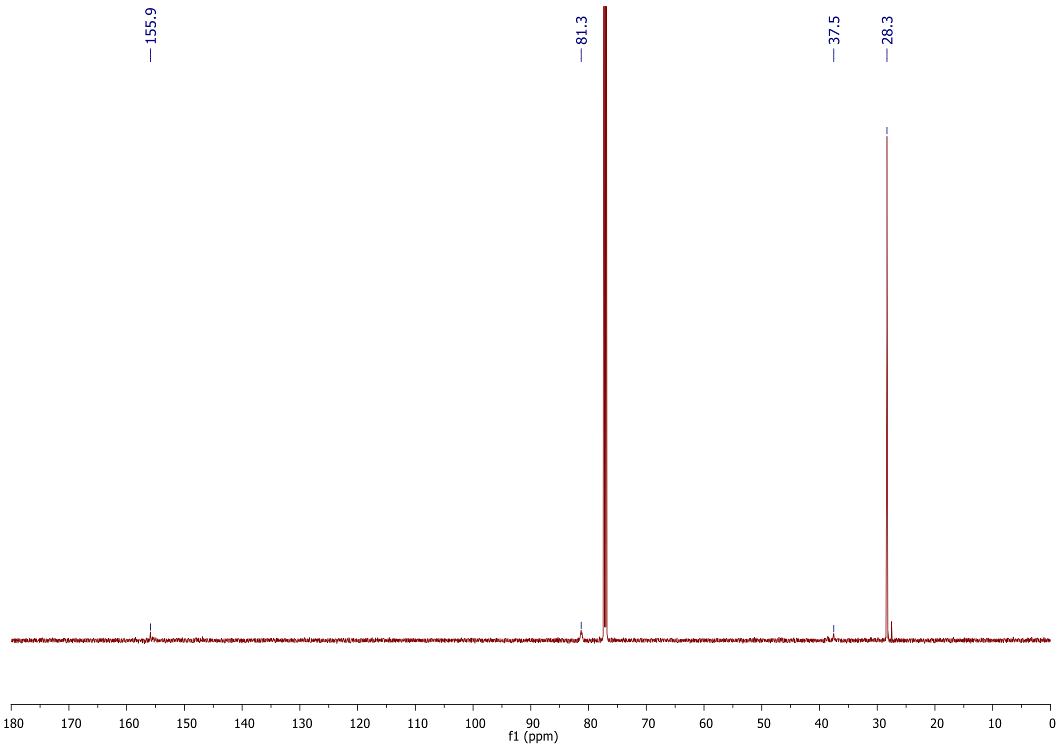
155.9
81.3
37.5
28.3
180
170
160
150
140
130
120
110
100
90
80
70
60
50
40
30
20
10
0
f1 (ppm)

Di-*tert*-butyl 1-(3-(tert-butoxy)-3-oxopropyl)-2-methylhydrazine-1,2-dicarboxylate[2] **2**

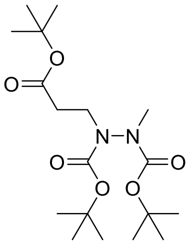

To a solution of di-*tert*-butyl 1-methylhydrazine-1,2-dicarboxylate (3.00 g, 12.2 mmol) in *t*-BuOH (5 mL), was added 10% NaOH (0.5 mL) and the reaction mixture stirred at 21 °C for 10 min. After this, *tert*-butyl acrylate (5.31 mL, 36.6 mmol) was added to the solution and the reaction mixture was heated at 60 °C for 24 h. Following this, the solvent was removed *in vacuo* and the crude residue was dissolved in EtOAc (150 mL) and washed with water (3 × 50 mL). The organic layer was then dried ($MgSO_4$) and concentrated *in vacuo*. Purification of the residue by flash column chromatography (0% to 20% EtOAc/petrol) afforded di-*tert*-butyl-1-(3-(*tert*-butoxy)-3-oxopropyl)-2-methylhydrazine-1,2-dicarboxylate **2** (2.24 g, 5.98 mmol, 49%) as a clear oil. ^{1}H NMR (600 MHz, $CDCl_3$, rotamers) δ 3.83–3.77 (m, 1H), 3.59–3.55 (m, 1H), 3.05–2.99 (m, 3H), 2.52-2.50 (m, 2H), 1.47–1.39 (m, 27H). ^{13}C NMR (150 MHz, $CDCl_3$, rotamers) δ 171.0 (C), 155.4 (C), 154.4 (C), 81.0 (C), 44.6 (CH_3), 36.6 (CH_2), 34.1 (CH_2), 28.3 (CH_3). IR (thin film) 2976, 2933, 1709 cm$^-$1. LRMS (ESI) 375 (100, $[M+H]^+$), 319 (30, $[M-C_4H_9+2H]^+$. HRMS (ESI) calcd for $C_{18}H_{35}N_2O_6$ $[M+H]^+$ 376.2524; observed 376.2516.

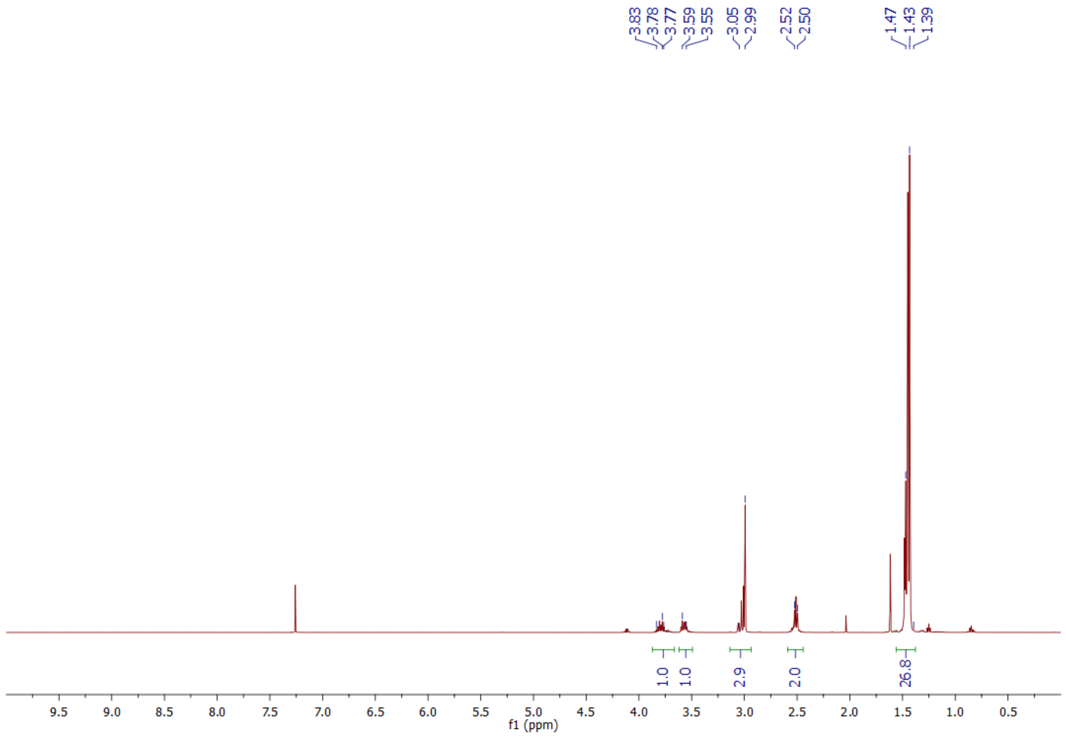
3.83
3.78
3.77
3.59
3.55
3.05
2.99
2.52
2.50
1.47
1.43
1.39
1.0
1.0
2.9
2.0
26.8
9.5
9.0
8.5
8.0
7.5
7.0
6.5
6.0
5.5
5.0
4.5
4.0
3.5
3.0
2.5
2.0
1.5
1.0
0.5
f1 (ppm)

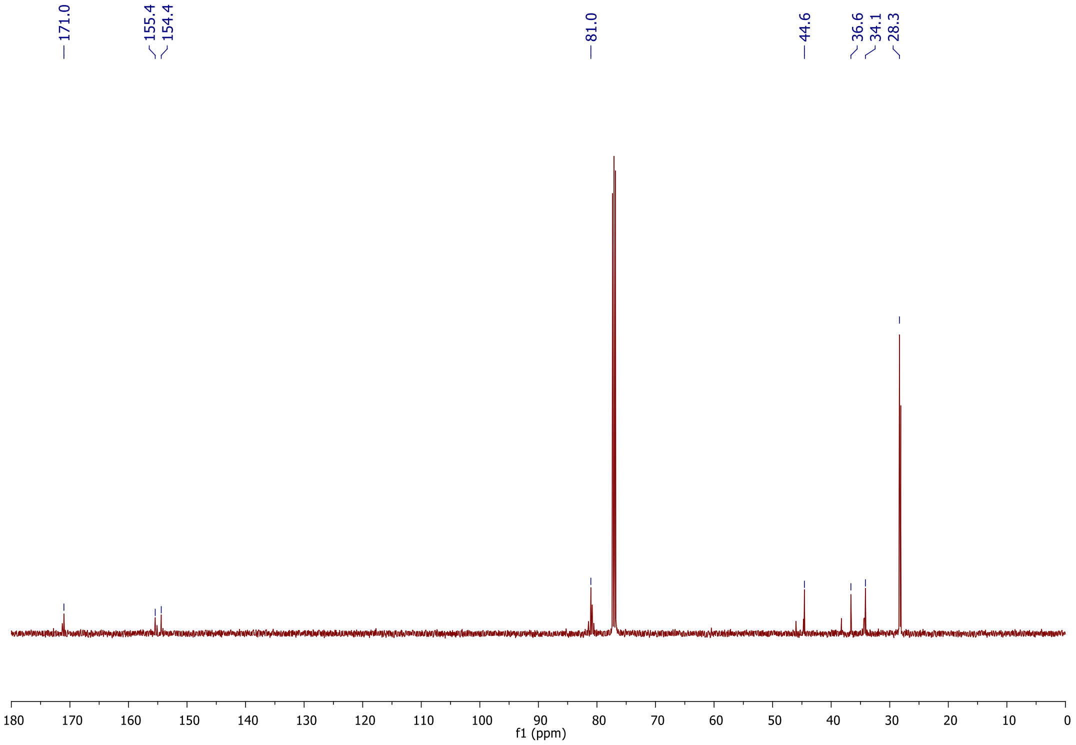
171.0
155.4
154.4
81.0
44.6
36.6
34.1
28.3
180
170
160
150
140
130
120
110
100
90
80
70
60
50
40
30
20
10
0
f1 (ppm)

3-(Bromo-2-methyl-3,6-dioxo-3,6-dihydropyridazin-1(2*H*)-yl)propanoic acid **3**

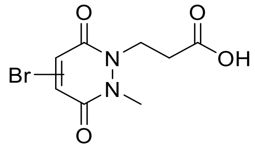

To a solution of di-*tert*-butyl-1-(3-(*tert*-butoxy)-3-oxopropyl)-2-methylhydrazine-1,2-dicarboxylate **2** (1.50 g, 4.01 mmol) in AcOH (20 mL) was added bromomaleic anhydride (0.41 mL, 4.41 mmol) and the reaction heated under reflux for 4 h. After this time, the reaction mixture was concentrated *in vacuo* with toluene co-evaporation (3 × 30 mL, as an azeotrope). The crude residue was then purified by flash column chromatography (0% to 10% MeOH/EtOAc (1% AcOH)) to afford an inseparable mixture of regioisomers 3-(4-bromo-2-methyl-3,6-dioxo-3,6-dihydropyridazin-1(2*H*)-yl)propanoic acid and 3-(5-bromo-2-methyl-3,6-dioxo-3,6-dihydropyridazin-1(2*H*)-yl)propanoic acid **3** (804 mg, 2.90 mmol, 72%) as a white solid. m.p. 142–145 °C. ^{1}H NMR (600 MHz, DMSO, regioisomers (1:1)) δ 7.59 (s, 1H), 7.58 (s, 1H), 4.28 (t, *J* = 7.4 Hz, 2H), 4.21 (t, *J* = 7.4 Hz, 2H), 3.58 (s, 3H), 3.50 (s, 3H), 2.62–2.57 (m, 4H). ^{13}C NMR (150 MHz, DMSO, regioisomers (1:1)) δ 172.0 (C), 171.9 (C), 155.4 (C), 155.1 (C), 153.5 (C), 153.2 (C), 135.8 (CH), 135.5 (CH), 132.8 (C), 132.3 (C) 42.7 (CH_2), 41.5 (CH_2), 34.2 (CH_3), 32.9 (CH_3), 31.8 (CH_2). IR (solid) 3058, 1722, 1619 cm^{-1}. LRMS (ESI) 277 (100, [M^{79}Br+H]$^{+}$), 279 (95, [M^{81}Br+H]$^{+}$), HRMS (ESI) calcd for $C_8H_{10}BrN_2O_4$ [M^{79}Br+H]$^{+}$ 276.9818; observed 276.9820.

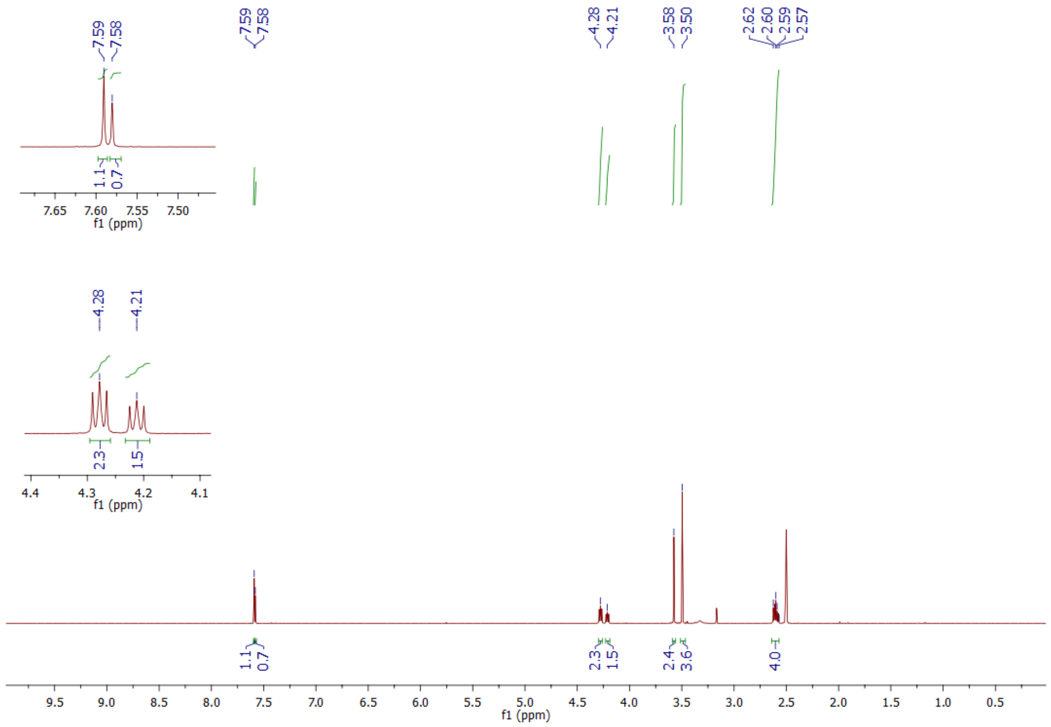
7.59
7.58
4.28
4.21
3.58
3.50
2.62
2.60
2.59
2.57
1.1
0.7
2.3
1.5
2.4
3.6
4.0
f1 (ppm)

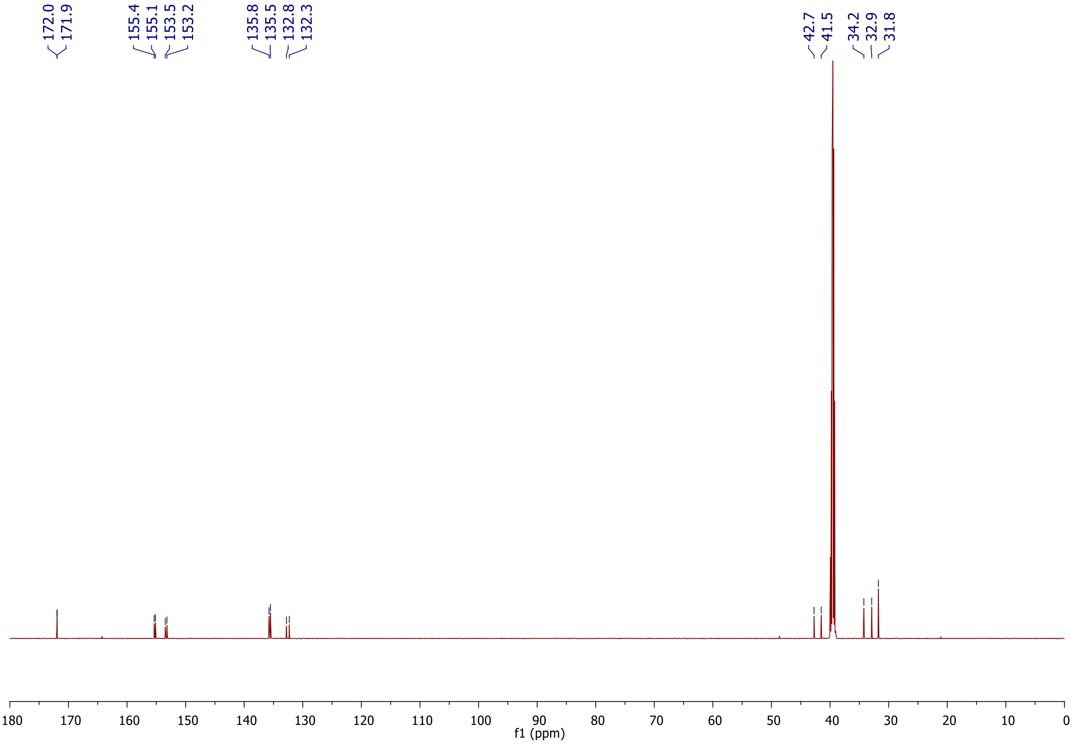
172.0
171.9
155.4
155.1
153.5
153.2
135.8
135.5
132.8
132.3
42.7
41.5
34.2
32.9
31.8
f1 (ppm)

2,5-Dioxopyrrolidin-1-yl 3-(4-bromo-2-methyl-3,6-dioxo-3,6-dihydropyridazin-1(2*H*)-yl)propanoate **4**

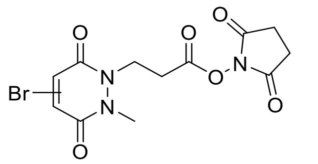

A solution of 3-(bromo-2-methyl-3,6-dioxo-3,6-dihydropyridazin-1(2*H*)-yl) propanoic acid **3** (1.20 g, 4.33 mmol), in THF (10 mL) was cooled to 0 °C and was added *N,N'*-dicyclohexylcarbodiimide (1.0 g, 4.85 mmol). The homogenous solution was then stirred at 0 °C for 30 min. After this time, was added *N*-hydroxysuccinimide (535 mg, 4.67 mmol) and the reaction was stirred at 21 °C for a further 16 h. The newly formed heterogenous mixture was then filtered and the filtrate concentrated *in vacuo*. Purification of the crude residue by flash column chromatography (50% to 100% EtOAc/petrol) afforded an inseparable mixture of regioisomers 2,5-dioxopyrrolidin-1-yl 3-(4-bromo-2-methyl-3,6-dioxo-3,6-dihydropyridazin-1(2*H*)-yl)propanoate and 2,5-dioxopyrrolidin-1-yl 3-(5-bromo-2-methyl-3,6-dioxo-3,6-dihydropyridazin-1(2*H*)-yl)propanoate **4** (1.0 g, 2.69 mmol, 62%) as a white powder. m.p. 140-145 °C. ^{1}H NMR (500 MHz, DMSO-d$_6$, regioisomers (1:1)) δ 7.34 (s, 1H), 7.31 (s, 1H), 4.42 (t, *J* = 7.2 Hz, 2H), 4.36 (t, *J* = 7.2 Hz, 2H), 3.62 (s, 3H), 3.55 (s, 3H), 3.06–3.01 (m, 4H), 2.79 (s, 8H). ^{13}C NMR (125 MHz, DMSO, regioisomers (1:1)) δ 170.0 (C), 166.7 (C), 155.4 (C), 155.2 (C), 153.5 (C), 153.3 (C), 135.9 (CH), 135.4 (CH), 133.0 (C), 132.1 (C), 41.7 (CH_2), 40.6 (CH_2), 34.3 (CH_3), 33.0 (CH_3), 28.5 (CH_2), 25.4 (CH_2). IR (solid) 2944, 1808, 1778, 1731, 1632, 1596 cm^{-1}. LRMS (ESI) 374 (100, [M^{79}Br+H]$^+$), 376 (95, [M^{81}Br+H]$^+$), HRMS (ESI) calcd for $C_{12}H_{12}BrN_3O_6$ [M^{79}Br+H]$^+$ 373.9988; observed 373.9979.

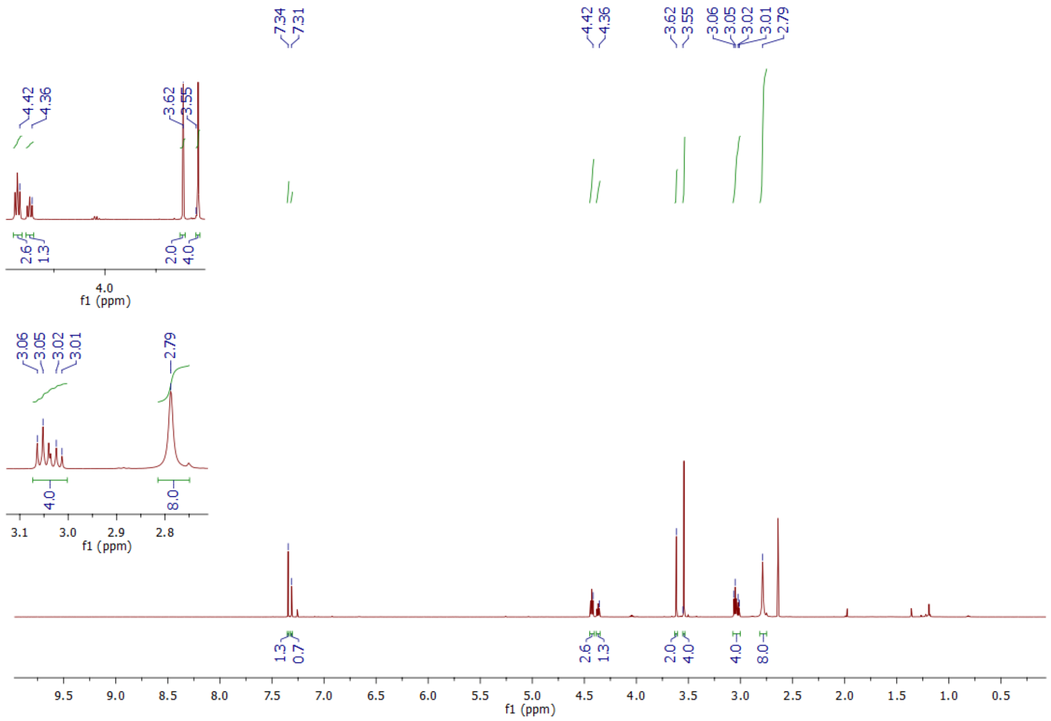
7.34
7.31
4.42
4.36
3.62
3.55
3.06
3.05
3.02
3.01
2.79
1.3
0.7
2.6
1.3
2.0
4.0
4.0
8.0
f1 (ppm)

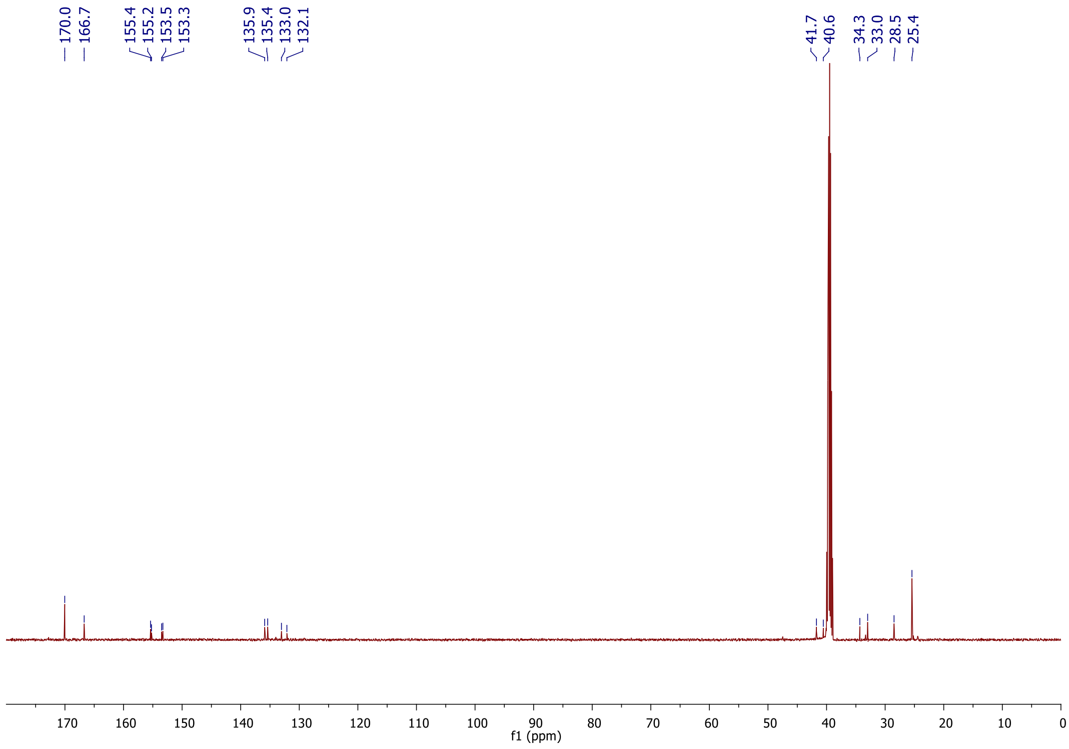
170.0
166.7
155.4
155.2
153.5
153.3
135.9
135.4
133.0
132.1
41.7
40.6
34.3
33.0
28.5
25.4
f1 (ppm)

((1*R*,8*S*,9*S*)-Bicyclo[6.1.0]non-4-yn-9-yl)methyl (2-(2-(2-(3-(4,5-dibromo-2-methyl-3,6-dioxo-3,6-dihydropyridazin-1(2*H*)-yl)propanamido)ethoxy)ethoxy)ethyl) carbamate **5**

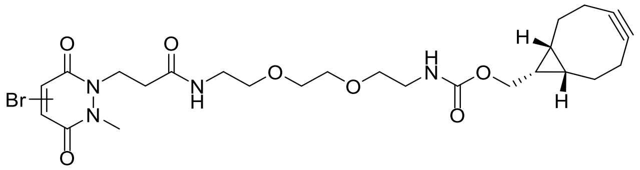

To a solution of 2,5-dioxopyrrolidin-1-yl 3-(bromo-2-methyl-3,6-dioxo-3,6-dihydropyridazin-1(2*H*)yl) propanoate **4** (50 mg, 0.110 mmol, pre-dissolved in MeCN (10 mL)), was added *N*-[(1*R*,8*S*,9*S*)-bicyclo[6.1.0]non-4-yn-9-ylmethyloxycarbonyl]-1,8-diamino-3,6-dioxaoctane (31 mg, 0.122 mmol) and the reaction mixture was stirred at 21 °C for 16 h. to afford an inseparable mixture of regioisomers ((1*R*,8*S*,9*S*)-Bicyclo[6.1.0]non-4-yn-9-yl)methyl (2-(2-(2-(3-(bromo-2-methyl-3,6-dioxo-3,6-dihydropyridazin-1(2*H*)-yl)propanamido)ethoxy)ethoxy)ethyl) carbamate **5** (42 mg, 0.07 mmol, 52%) as a yellow oil: ^{1}H NMR (600 MHz, $CDCl_3$, regioisomers (1:1)) δ 7.86 (s, 0.5H), 7.80 (s, 0.5H), 7.38-7.36 (m, 2H), 6.42-6.39 (m, 1H), 5.81-5.78 (m, 1H), 5.33 (s, 0.5H), 5.27 (m, 0.5H), 4.44 (t, *J* = 7.0 Hz, 2H), 4.37 (t, *J* = 7.0 Hz, 2H), 4.18-4.15 (m, 4H), 3.73-3.67 (m, 3H), 3.66-3.36 (m, 27H), 2.63-2.61 (m, 4H), 2.32-2.21 (m, 12H), 1.65-1.52 (m, 4H), 1.42-1.31 (m, 2H), 0.96-0.93 (m, 4H). ^{13}C NMR (150 MHz, $CDCl_3$, regioisomers (1:1)) δ 171.5 (C),168.9 (C), 156.9 (C), 155.9 (C), 139.4 (C), 136.1 (C), 135.7 (C), 98.9 (C), 70.7 (CH_2), 70.3 (CH_2), 69.7 (CH_2), 62.3 (CH_2), 60.6 (CH_2), 44.2 (CH_2), 43.4 (CH_2), 40.8 (CH_2), 39.5 (CH_2), 34.7 (CH_3), 34.0 (CH_2), 33.4 (CH_2), 33.1 (CH_2), 29.2 (CH_2), 21.6 (CH_2), 21.0 (CH_2), 20.2 (CH_2), 17.9 (CH), 14.3 (CH). IR (thin film) 3331, 2989, 2857, 1715, 1645, 1572, 1534 cm^{-1}. LRMS (ESI) 583 (95, $[M^{79}Br+H]^+$), 585 (100, $[M^{81}Br+H]^+$), HRMS (ESI) calcd for $C_{25}H_{35}BrN_4O_7$ $[M^{79}Br+H]^+$ 583.1714; observed 583.1763.

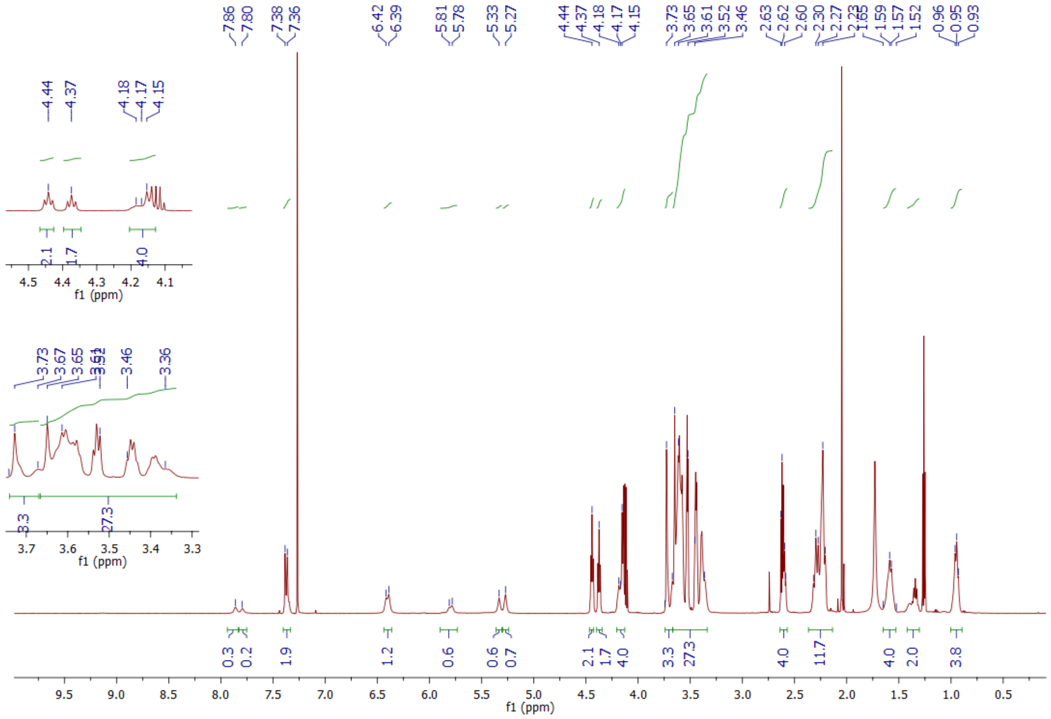
7.86
7.80
7.38
7.36
6.42
6.39
5.81
5.78
5.33
5.27
4.44
4.37
4.18
4.17
4.15
3.73
3.65
3.61
3.52
3.46
2.63
2.62
2.60
2.30
2.27
2.23
1.65
1.59
1.57
1.52
0.96
0.95
0.93
0.3
0.2
1.9
1.2
0.6
0.6
0.7
2.1
1.7
4.0
3.3
27.3
4.0
11.7
4.0
2.0
3.8
f1 (ppm)
2.1
1.7
4.0
3.73
3.67
3.65
3.61
3.36
3.3
27.3

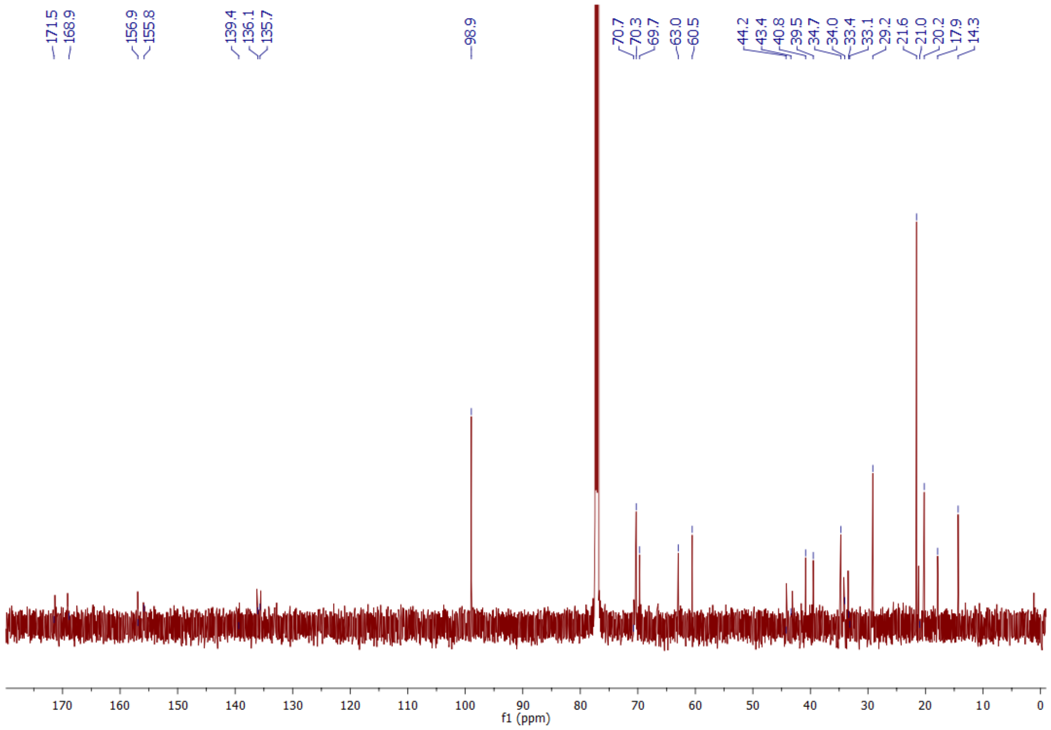
171.5
168.9
156.9
155.8
139.4
136.1
135.7
98.9
70.7
70.3
69.7
63.0
60.5
44.2
43.4
40.8
39.5
34.7
34.0
33.4
33.1
29.2
21.6
21.0
20.2
17.9
14.3
f1 (ppm)

V_{NAR} E4 Conjugation data

V_{NAR} E4 **6**

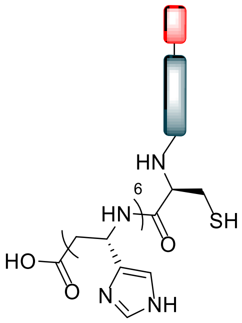

E4 ACA was diluted to 33.5 µM, 100 µL, in PBS pH = 7.4 and then reduced at room temperature for 5 h with TCEP (2 µL, 16.75 mM, 10 eq. in PBS pH = 7.4). Successful reduction was confirmed by LC-MS. Expected mass: 12753; Observed mass: 12756.

Non-deconvoluted and deconvoluted MS data for V_{NAR} E4 6

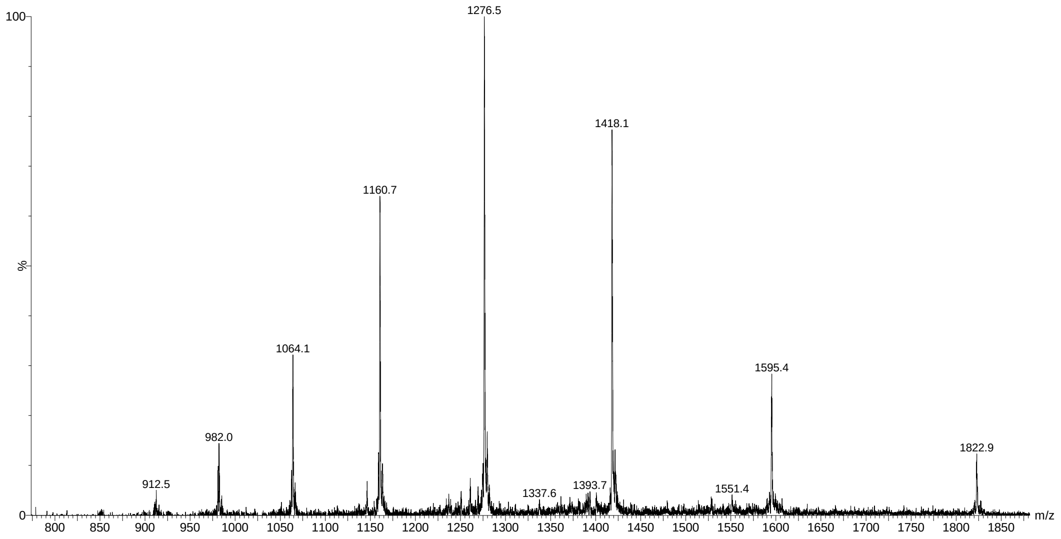

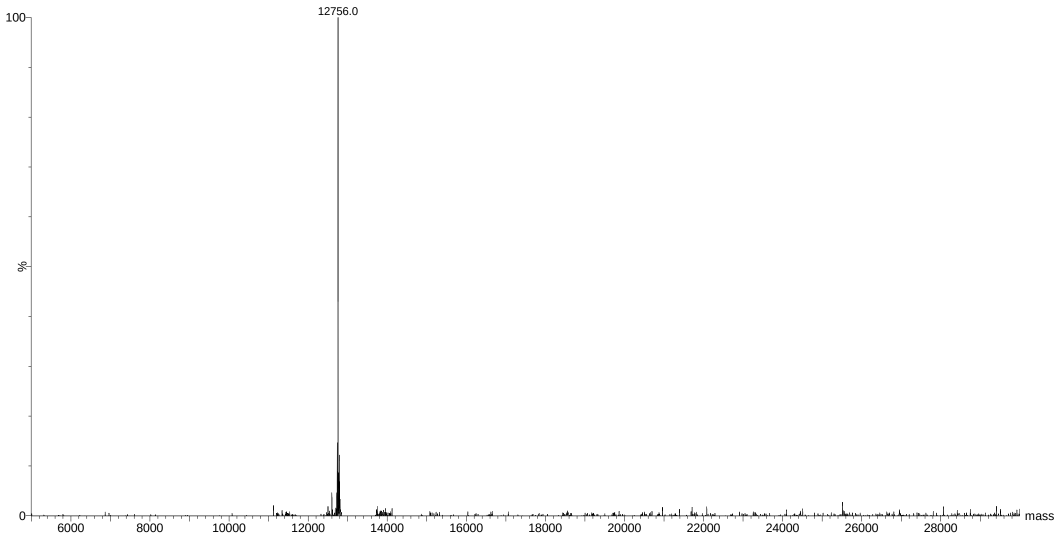

V_{NAR} E4 conjugate **7**

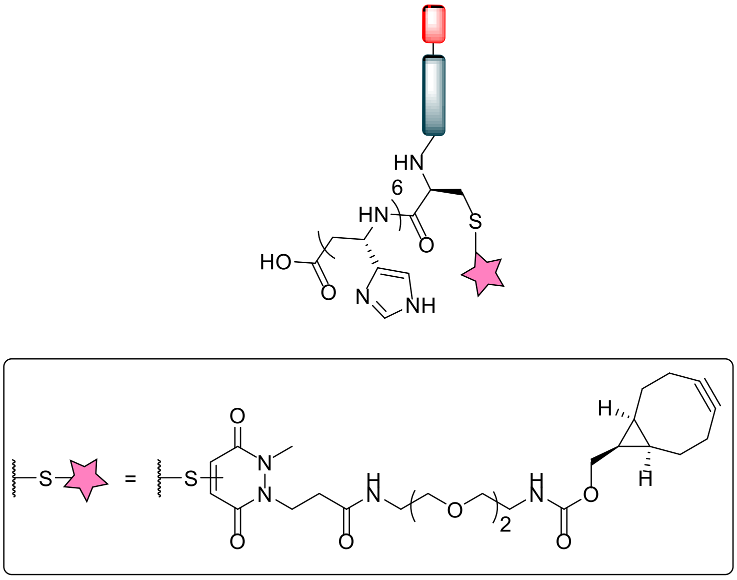

V_{NAR} E4 **6** was diluted to 33.5 µM, 100 µL, in PBS, pH = 7.4 and then reduced at room temperature for 5 h with TCEP (2 µL, 16.75 mM, 10 eq. in PBS pH = 7.4). After this time, the clone was reacted with PD **5** (0.85 µL, 80 mM, 20 eq. in DMSO), and incubated at 21 °C for 16 h.

Purification

Although some of the attempted purification methods (SEC, de-salting columns, 5kDa ultrafiltration), afforded clean conjugates, they resulted in unpredictable and frequently poor yields (most attempts recorded below 20% protein recovery). Fortunately, it was found that by diminishing the concentration of DMSO in the sample by preparing a more concentrated sample of PD **5**, it was possible to achieve recoveries of over 80% whilst repeatedly removing excess small molecules *via* 5,000 MWCO ultrafiltration (*i.e.* high concentrations of DMSO caused damage to the filters, permitting the protein to pass through). Thus, the excess reagents were then removed by repeated diafiltration into fresh buffer using VivaSpin sample concentrators (GE Healthcare, 5,000 MWCO). Successful conjugation was confirmed by LC-MS. Expected mass: 13,256 Da. Observed mass: 13,258 Da.

Non-deconvoluted and deconvoluted MS data for V_{NAR} E4 conjugate 7

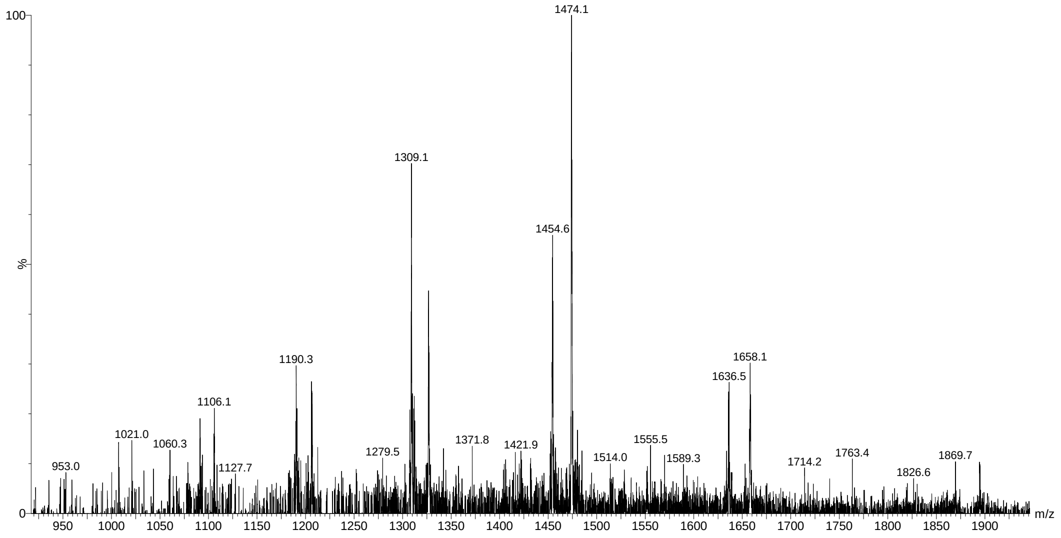

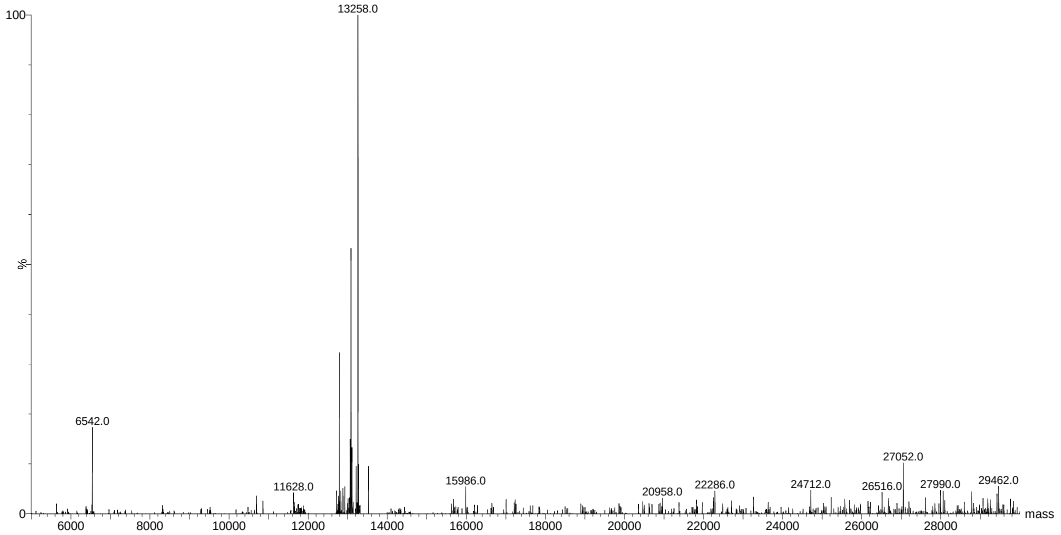

V_{NAR} Alexafluor **8**

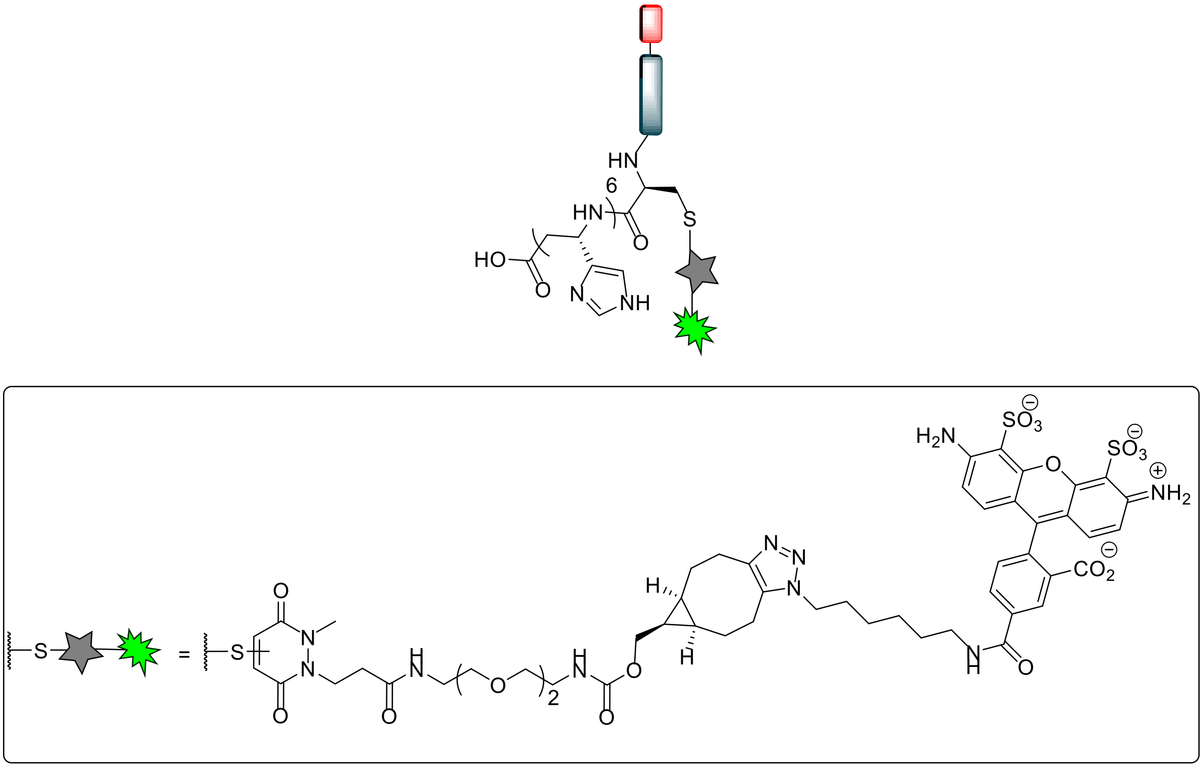

To a solution of V_{NAR} conjugate **7** (50 µL, 33.5 µM) in PBS (pH= 7.4) was added Alexafluor®-488-N_3 (0.85 µL, 20 mM in DMSO, 10 eq.) and the reaction mixture incubated at 21 °C for 6 h. The excess reagents were then removed by repeated diafiltration into fresh buffer using VivaSpin sample concentrators (GE Healthcare, 5,000 MWCO). Successful conjugation was confirmed by SDS-PAGE and LC-MS. Expected mass: 13,916 Da. Observed mass: 13,918 Da.

Non-deconvoluted and deconvoluted MS data for V_{NAR} Alexafluor 8

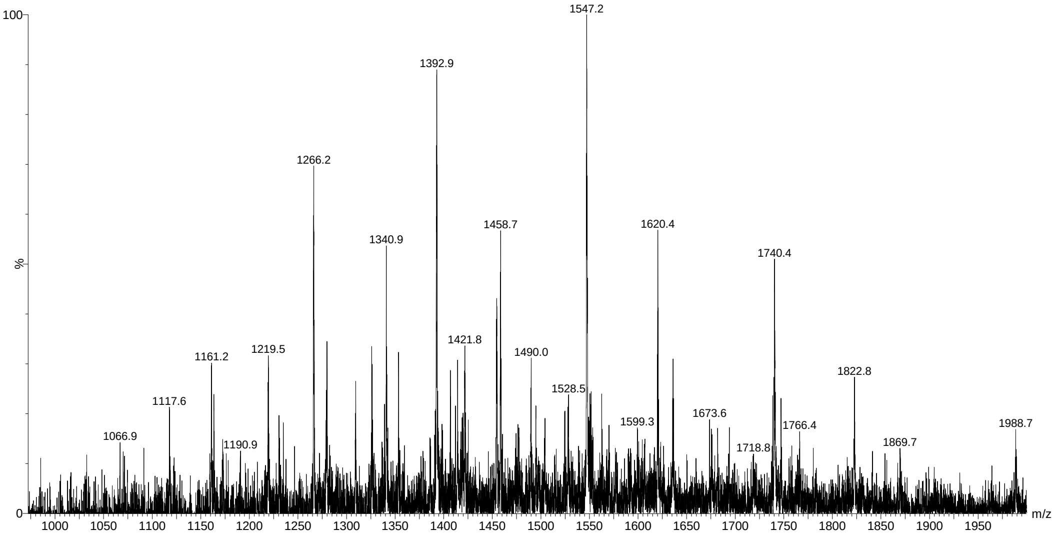

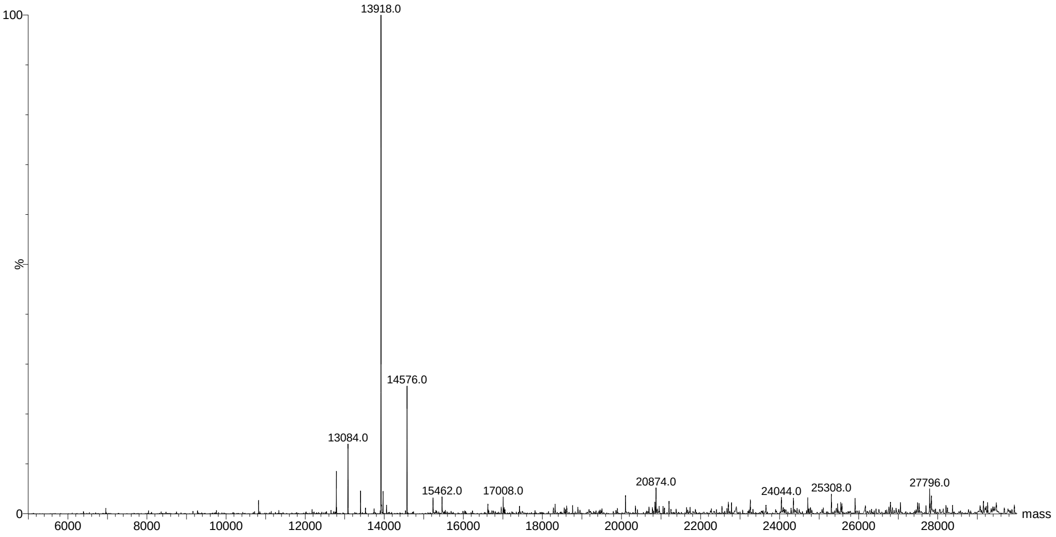

Additional Figures

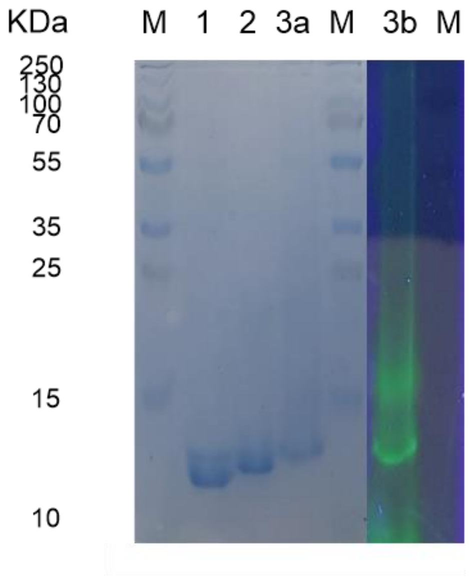

Figure S2 SDS-PAGE for $\mathbf{V_{NAR}}$ E4 conjugates. M) Molecular weight marker; **1a)** Stained V_{NAR} E4 **6**; **2a)** Stained V_{NAR} conjugate **7**; **3a)** Stained V_{NAR} Alexafluor **8**; **3b)** V_{NAR} Alexafluor **8** under UV light.

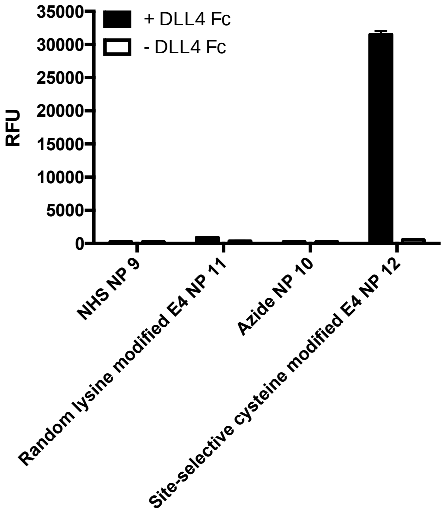

Figure S3 Random lysine modified E4 NP **11** and site-selective cysteine modified E4 NP **12** were formulated with equimolar amounts of V_{NAR} conjugated per milligram of polymer. Binding of these nanoformulations and the corresponding blank NP controls (500 µg polymer/mL) to DLL4 was analysed by modified ELISA. Data expressed as mean ± SEM.

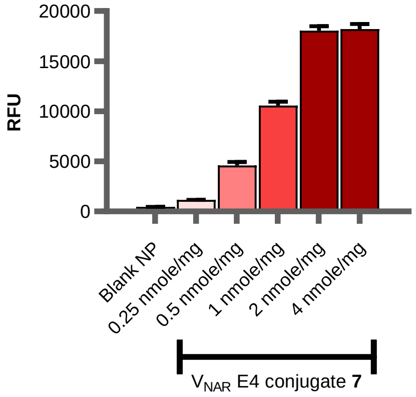

Figure S4 Various amounts of V_{NAR} conjugate **7** were incubated with fluorescently labelled NP composed of a 75:25 blend of PLGA-502H:PLGA-PEG-azide. Binding of these nanoformulations and the corresponding blank NP control (500 µg polymer/mL) to DLL4 was analysed by modified ELISA. Input amounts of V_{NAR} conjugate **7** are annotated as nanomoles of V_{NAR} per milligram of polymer. Data expressed as mean ± SEM.

Nanoformulation	Diameter (nm)[a]	Polydispersity index[a]	Zeta potential (mV)[a]	V_{NAR} conjugated (pmole/mg polymer)[a]	Conjugation efficiency (%)[a]
NHS NP **9**	191.5 ± 5.3	0.11 ± 0.04	-1.6 ± 3.1	-	-
Random lysine modified E4 NP **11**	197.8 ± 5.1	0.12 ± 0.05	0.7 ± 4.8	26.8 ± 19.5	10.7 ± 7.8
Azide NP **10**	194.0 ± 2.5	0.07 ± 0.04	1.9 ± 4.3	-	-
Site-selective cysteine modified E4 NP **12**	214.2 ± 13.3	0.10 ± 0.04	0.7 ± 4.8	91.4 ± 50.0	36.6 ± 20.0

Table S1 Characterisation of nanoformulations. [a] Data expressed as mean ± standard deviation.

1 M. K. Greene, D. A. Richards, J. C. F. Nogueira, K. Campbell, P. Smyth, M. Fernández, C. J. Scott and V. Chudasama, *Chem. Sci.*, 2018, **9**, 79–87.

2 C. Bahou, D. A. Richards, A. Maruani, E. A. Love, F. Javaid, S. Caddick, J. R. Baker and V. Chudasama, *Org. Biomol. Chem.*, 2018, **16**, 1359–1366.
